# Leave Nothing Behind in Coronary Intervention: Time to Move From Promise to Proof

**DOI:** 10.1002/clc.70228

**Published:** 2025-12-02

**Authors:** Hasnain Wajeeh Saqib, Talha Khan, Rehman Bashir, Tazeem Hayat, Areesha Ishfaq Ahmed, Ayesha Tariq

**Affiliations:** ^1^ Riphah International University Islamic International Medical College Rawalpindi Pakistan

We read with great interest the recent article by Meunier et al. [1] reporting the 3‐year follow‐up of a prospective all‐comers observational study evaluating a stentless percutaneous coronary intervention (PCI) strategy with drug‐coated balloons (DCB). The authors are to be commended for providing one of the most comprehensive long‐term datasets on this approach. Their findings, particularly the low rate of target lesion revascularization (TLR) in the DCB‐only cohort (2.6%) compared with the bailout drug‐eluting stent (DES) group (6%), underscore the feasibility of a “leave nothing behind” strategy in contemporary practice.

The rationale for DCB angioplasty rests on avoiding the long‐term complications of permanent metallic scaffolds, including in‐stent restenosis, neoatherosclerosis, and late thrombosis [2]. The durability of outcomes over 3 years in an unselected population provides important reassurance for interventional cardiologists considering wider adoption of a DCB‐first approach.

However, the absence of randomization precludes firm causal inference. The differences in TLR‐free survival (*p* = 0.016) may reflect lesion complexity or operator selection bias rather than an actual treatment effect. Randomized controlled trials directly comparing stentless PCI with current‐generation DES are required to establish durability, safety, and generalizability [3, 4]. Such studies should also examine endpoints beyond TLR, including myocardial infarction, late thrombosis, and patient‐reported outcomes.

Notably, the introduction of the “metal index” serves as a quantitative marker of stent burden. The observation that higher indices were associated with worse outcomes highlights their potential as a prognostic tool. Future research should validate the metal index in diverse cohorts and determine whether it can guide clinical decision‐making, particularly when hybrid strategies necessitate bailout stenting.

Patient and lesion selection also remain critical considerations. While small‐vessel disease, bifurcations, and high‐bleeding‐risk subsets have been the traditional focus of DCB angioplasty [5], the favorable long‐term outcomes reported here suggest that the potential scope of patients who may benefit could be broader. Rigorous comparative studies, including registry‐based analyses, will be essential to delineate these populations more precisely.

In summary, Meunier et al. provide compelling evidence that reinforces the biological and clinical appeal of a stentless PCI strategy, advancing the dialogue on the “leave nothing behind” paradigm. At the same time, their work should be viewed as a call to action: large‐scale randomized trials are urgently needed to validate these observational insights, to explore the clinical utility of the metal index, and to define the optimal patient subsets for DCB‐first strategies.

## Author Contribution

**Hasnain Wajeeh Saqib:** writing – original manuscript. **Talha Khan:** writing – original manuscript. **Rehman Bashir:** writing – original manuscript. **Tazeem Hayat:** writing – original manuscript. **Areesha Ishfaq Ahmed:** writing – original manuscript. **Ayesha Tariq:** writing – original manuscript.

## Ethics Statement

The authors have nothing to report.

## Conflicts of Interest

The authors declare no conflicts of interest.

## Data Availability

The authors have nothing to report.
